# Multiple sarcomatoid carcinoma of the small intestine with perforation as the initial symptom: case report and review of the literature

**DOI:** 10.3389/fonc.2024.1477951

**Published:** 2024-12-24

**Authors:** Jixin Fu, Qingbin Kong, Xin Sui, Xinjian Wang

**Affiliations:** Department of Gastrointestinal Surgery, Weihai Central Hospital, Qingdao University, Weihai, Shandong, China

**Keywords:** small intestine, sarcomatoid carcinoma, gastrointestinal perforation, case report, biphasic tumor

## Abstract

**Background:**

Sarcomatoid carcinoma of the small bowel is an exceedingly rare gastrointestinal tumor characterized by a biphasic cellular pattern of epithelioid and mesenchymal-like cells. Due to its rarity and non-specific clinical presentation, it is frequently misdiagnosed, and there is a lack of standardized management guidelines. We report a case of multiple sarcomatoid carcinoma of the small intestine, presenting initially with gastrointestinal perforation. Additionally, we conducted a comprehensive review and analysis of the clinical manifestations, immunohistological characteristics, and prognostic factors associated with small intestinal sarcomatoid carcinoma, aiming to enhance diagnostic accuracy and therapeutic strategies for this rare malignancy.

**Case presentation:**

An 82-year-old man was admitted with a 1-week history of abdominal pain, exacerbated by the onset of fever in the last 24 hours. Abdominal CT revealed thickening of the small intestinal wall and free gas within the mesenteric space, indicating gastrointestinal perforation. Emergency surgery identified multiple tumors in the small intestine, accompanied by perforation. Postoperative pathology confirmed the diagnosis of sarcomatoid carcinoma of the small intestine.

**Conclusion:**

We report a rare case of sarcomatoid carcinoma of the small intestine and conduct a thorough literature review to offer new insights into its diagnosis, treatment, and prognosis. This highly malignant tumor, predominantly found in the jejunum and ileum, is characterized by high recurrence and metastasis rates, leading to a poor prognosis. Notably, postoperative radiotherapy does not improve outcomes. Abdominal CT is highly sensitive for detecting small bowel tumors but cannot confirm SCA due to its nonspecific imaging features. In contrast, small enteroscopy or capsule endoscopy offers greater diagnostic clarity. Increased awareness among clinicians is crucial for early detection and intervention.

## 1 Introduction

The small bowel is a highly active organ, responsible for up to 90% of nutrient absorption and comprising 75% of the length of the gastrointestinal tract (GIT) (1). Despite this activity and high rate of cellular renewal, small bowel tumors are rare, accounting for only 1%–3% of all gastrointestinal (GI) cancers (2). The most common pathological types of small bowel tumors include adenocarcinomas, neuroendocrine tumors, lymphomas, and gastrointestinal mesenchymal tumors, while primary sarcomatoid carcinoma of the small bowel (SCA), which is composed of malignant epithelial and mesenchymal components, is extremely rare (3, 4). The pathogenesis of SCA remains unclear. The collision theory is a prominent hypothesis that suggests two different types of tumor cells originate from the mesenchyme and epithelium, respectively (5). Recent genome sequencing studies suggest a more plausible theory that sarcoma-like and carcinoma-like cells in sarcomatoid carcinoma share a common clonal origin (6, 7). SCA has been identified in various organs, including the lungs, uterus, salivary glands, and thyroid gland (8, 9). Although Dikman SH first described small bowel SCA, using the term “enteroblastoma” in 1973 (10), reports of sarcomatoid carcinoma of the small intestine remain limited to this day.

The incidence of SCA of the small intestine is estimated at 0.5 to 0.8 cases per 100,000 people per year (11–13). The disease shows a higher prevalence in males, with a male-to-female ratio of 1.5:1 (14). Although the risk factors remain unclear, some reports suggest a possible association with long-term local enteritis (15). Common symptoms include anemia and non-specific signs such as abdominal pain, nausea, vomiting, obstruction, weight loss, gastrointestinal bleeding, palpable abdominal masses, and fatigue (12, 15, 16). The occurrence of multiple sarcomatoid carcinomas of the small intestine presenting with perforation as the initial symptom is exceedingly rare. We report a case of this presentation and conduct a comprehensive literature review to enhance understanding of this unusual disease.

## 2 Case presentation

A 82 old man presented with a week-long history of abdominal pain, which acutely worsened with the onset of fever for one day. His medical history included hypertension, managed with oral timosartan.

The patient experienced paroxysmal abdominal pain around the umbilicus without obvious triggers one week ago. There was no radiating pain to the shoulder or back, no nausea or vomiting, no chest tightness or breath-holding, and no chills or fever. The patient remained untreated. One day ago, the abdominal pain suddenly worsened, becoming persistent and spreading across the entire abdomen, accompanied by a fever peaking at 37.7°C. The patient urgently sought care at our hospital’s Emergency Department.

Physical Examination: The patient was mentally clear and cooperative during the examination. The abdomen was flat and soft, with no visible gastrointestinal distention or peristaltic waves. Abdominal muscles were tight, with significant tenderness and rebound pain in the lower abdomen. No palpable masses were detected, and there was no significant percussion pain in the liver or kidney areas. Intestinal sounds were weak, occurring twice per minute.

**Figure 1:**
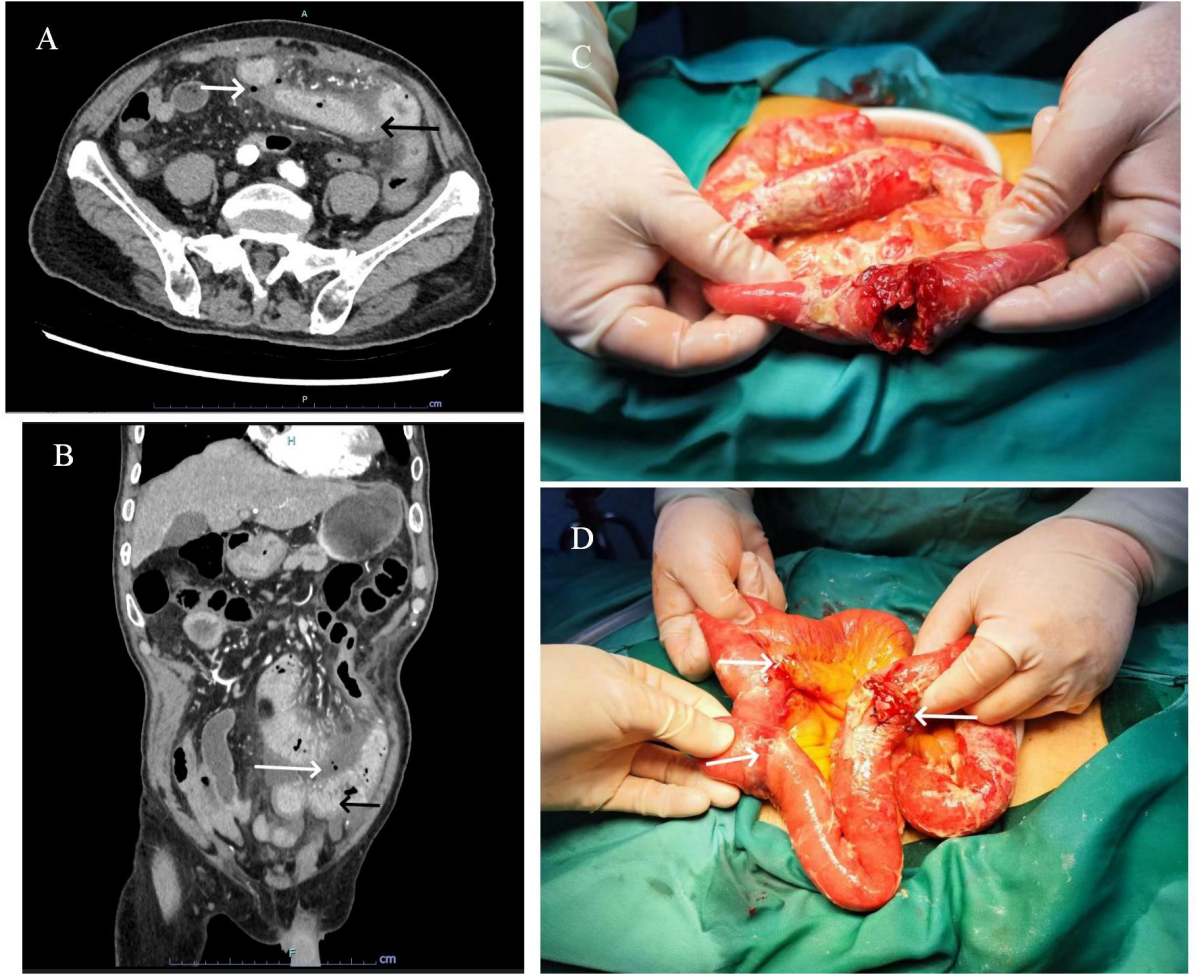
Transverse **(A)** and sagittal **(B)** enhanced CT images of the abdomen reveal a thickened, abnormally enhanced bowel wall (black arrows) and free air bubbles in the mesenteric space (white arrows). A cauliflower-shaped mass with a central perforation was identified in the proximal small intestine **(C)**. Additionally, two sclerotic nodules were observed distally, invading the plasma membrane layer **(D)**.

Laboratory Tests: The patient’s laboratory results revealed a leukocyte count of 21.88 × 10^9^/L (reference interval 3.5-9.5×10^9^/L), a neutrophil percentage of 94.5% (reference interval 40-75%), C-reactive protein at 80.91 mg/L (reference interval 0-3 mg/L), and calcitoninogen at 3.09 ng/ml (reference interval <0.06 ng/mL). Liver function, renal function, and coagulation tests were all within normal limits. The electrocardiogram was normal. A chest Computed Tomography (CT) showed a mass in the lower lobe of the right lung. An abdominal enhanced CT scan indicated thickening and abnormal enhancement of the wall of the middle and lower intestines, with free air bubbles in the mesenteric space (**Figures 1A, B**).

**Figure 2:**
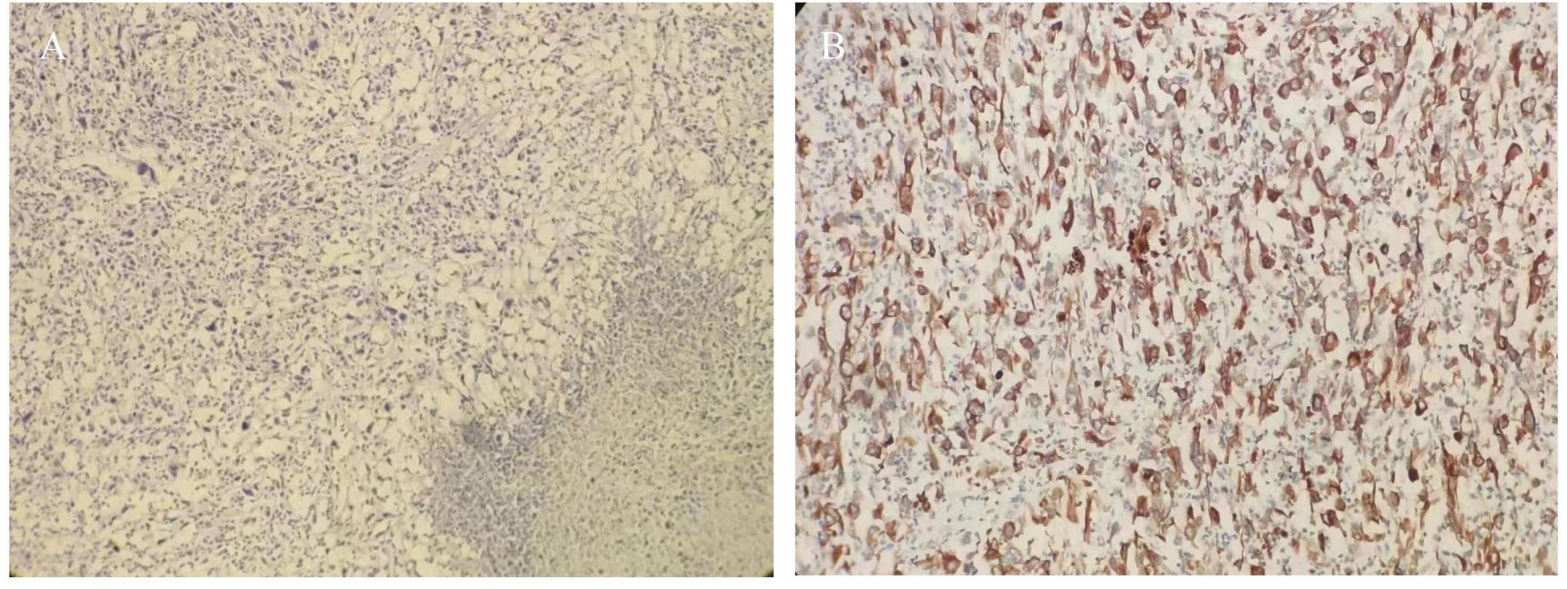
**(A)** HE staining tumor cells were short spindle-shaped and ovoid (HE ×100); **(B)** Immunohistochemistry cytokeratin (CK) was diffusely positive (×200).

In consideration of gastrointestinal perforation, emergency laparoscopic exploration was performed. Intraoperatively, a significant amount of yellowish-white pus and intestinal contents were observed in the abdominal cavity. A cauliflower-like mass, approximately 5×4 cm in size, was identified in the small intestine, located approximately 20 cm from the ligament of Treitz. Central perforation with overflow of intestinal fluid was noted at the site (**Figure 1C**). Additionally, two hard nodules were detected in the distal part of the perforation, spaced at intervals of 20 and 30 cm, respectively, with invasion of the serosal (**Figure 1D**). The intraoperative diagnosis was multiple small bowel tumors with perforation. Subsequently, small bowel tumor resection was performed. Following surgery, the patient received anti-infective treatment, gastric mucosa protection, and parenteral nutritional support. Postoperative pathological examination revealed multiple SCA of the small intestine, with three foci of the mass identified, the largest measuring 6×3 cm. The mass exhibited invasion of the serosal, with evidence of vascular invasion. No tumors were observed in the surgical incision lines at both ends, nor in the peripheral intestinal lymph nodes (0/14). The tumor was staged at the pathological stage of pT4N0Mx [The eighth edition of the American Joint Committee on Cancer (AJCC)]. Immunohistochemical analysis revealed the following: cytokeratin (CK) (+), Epithelial membrane antigen (EMA) (weak +), SOX10 (-), S-100 (-), Melan-A(-), Melanoma Marker (HMB45) (-), CD68(-), Syn(-), CD34(-), ERG(-), DOG1(-), Vimentin (+), CD21 (-), Desmin (-), CD4 5(partial+), myeloperoxidase (MPO) (-) (**Figures 2A, B**).

Following the postoperative pathological findings, a multidisciplinary discussion was organized. It was recommended that the patient undergo puncture biopsy of the right lung mass to clarify its nature and consider further chemotherapy treatment in the oncology department. However, the patient declined this recommendation for personal reasons and was discharged on the 9th postoperative day. On follow-up after 2 months, the patient had lost 5 kg and abdominal CT showed extensive metastases in the patient’s abdominal cavity. The patient ultimately passed away 3 months postoperatively due to widespread tumor metastases and multiple organ failure.

## 3 Discussion

Despite the small intestine comprising 75% of the gastrointestinal tract’s length and 90% of its absorptive area, tumors in this region are exceedingly rare. SCA of the small bowel is a histologically distinct type, featuring a high prevalence of spindle and epithelioid cells. This tumor can occur in various organs and systems, including the digestive and respiratory tracts, thyroid gland, breast, and salivary glands. The sarcomatoid component in small bowel sarcomatoid carcinoma may present as fibrosarcoma, malignant fibrous histiocytoma, rhabdomyosarcoma, hemangiosarcoma, or chondrosarcoma (17). Small bowel SCA is often asymptomatic or presents with nonspecific symptoms, such as abdominal pain, melena, intestinal obstruction, and jaundice. These manifestations overlap with conditions like metastatic sarcomatoid carcinoma, malignant melanoma, gastrointestinal stromal tumors (GIST), and familial adenomatous polyposis-associated carcinoma, making diagnosis particularly challenging. Unlike other small bowel tumors, SCA is poorly responsive to radiotherapy, and nearly 50% of patients present with metastases at diagnosis, leading to a poor prognosis with high rates of recurrence and metastasis-related mortality (18, 19). Improving clinical awareness, enhancing early detection, and prioritizing timely surgical intervention are crucial to improving patient outcomes.

To conduct a comprehensive literature review on SCA of the small intestine, we searched PubMed (https://www.ncbi.nlm.nih.gov/pubmed), Wanfang Database (http://www.wanfangdata.com.cn/index.html), and the China Knowledge Network (CNKI; http://kns.cnki.net/kns/brief/default_result.aspx) using MeSH terms and keywords such as “sarcomatoid carcinoma,” “metaplastic carcinoma,” “spindle cell carcinoma,” and “pleomorphic carcinoma.” We also screened reference lists to identify additional relevant studies and extracted data using standardized forms. Our review identified approximately 43 cases of SCA of the small intestine (**Table 1**). To our knowledge, this is one of the most extensive literature reviews on this rare condition.

**Table 1:** Sarcomatoid carcinoma (SCA) of the small bowel reported in the literature.

	Age (year)	Gender	Presentation	Diagnostic method	Single or multiple	Tumor size* (cm)	Location	Immunohistochemistry (+)	Metastasis	Treatment	Prognosis (month)	Ref.
2002	56	Male	Right supraclavicular nodules	angiography	Single	9.2×5.2×2.3	jejunum	Cytokeratins; Vimentin; EMA	No	surgery	3/dead	(25)
2024	61	Male	Abdominal pain and bloating	CT scan	multiple	8×5×3.5	jejunum	Cytokeratins; Vimentin; CD34	Yes	surgery	5/dead	(2)
1991	57	Male	melaena	NA	Single	14×10× 2	jejunum	Vimentin; Cytokeratins; EMA	No	surgery	6/alive	(26)
1991	63	Male	weight loss	NA	Single	6×4.5×2	ileum	Vimentin;Cytokeratins; Desmin	No	surgery	39/alive	(26)
1991	45	Male	Obstruction	NA	Single	3×32	ileum	Vimentin; Cytokeratins	Yes	surgery	0.13/dead	(26)
2021	54	Male	Obstruction	CT scan	multiple	2.6×2.6	Jejunum+ileum	Vimentin; Cytokeratins; CD34; CD68	No	Surgery+chemotherapy	2/dead	(23)
1996	72	Male	NA	NA	Single	NA	duodenum	Vimentin; Cytokeratins	No	surgery	7/dead	(27)
1996	68	Female	abdominal pain	NA	Single	5×4×6	ileum	Vimentin; Cytokeratins; EMA; α-1 Antichymotypsin; α-1-antitrypsin	No	surgery	7/alive	(27)
1996	75	Male	abdominal pain	CT scan	Single	3×4×5	ileum	Vimentin; Cytokeratins; EMA; α-1 Antichymotypsin; α-1-antitrypsin	No	surgery	4/alive	(27)
2009	55	Male	weight loss	CT scan	Single	8×7	jejunum	Cytokeratins; EMA	No	Surgery+chemotherapy	9.43/dead	(28)
2022	61	Male	abdominal pain and weight loss	CT scan	Single	7.8×7.0×7.1	duodenum	EMA; S100	Yes	chemotherapy	NA	(29)
1999	53	Male	bleeding	CT scan	Single	13×12×3	ileum	Cytokeratins; EMA	Yes	Surgery+chemotherapy	2/dead	(30)
2011	51	Female	anemia and abdominal pain	X-ray	Single	8×8	jejunum	Vimentin; Cytokeratins; CD68	No	Surgery+chemotherapy	2.75/dead	(31)
2022	73	Female	abdominal pain and weight loss	CT scan	Single	NA	NA	Vimentin; Cytokeratins; EMA; PD-L1	Yes	Surgery+immunotherapy	21/alive	(32)
2016	58	Female	Obstruction	MRI	Single	3×3×2	ileum	Vimentin; Cytokeratins; Ki67	No	Surgery+chemotherapy	6/alive	(12)
2015	56	Male	abdominal pain and weight loss	Enteroscopy	Single	14×14	jejunum	Vimentin; Cytokeratins; EMA	Yes	Surgery+chemotherapy	7/alive	(17)
2004	55	Male	Bleeding and weight loss	Enteroscopy	Single	7.5×6.3×2.5	jejunum	Vimentin; Cytokeratins; EMA; CD30	No	Surgery	8/dead	(11)
1989	52	Female	anemia and abdominal pain	NA	multiple	8×4	jejunum	Vimentin; Cytokeratins; EMA	Yes	Surgery+chemotherapy	7/dead	(33)
1989	56	Male	abdominal pain and weight loss	NA	multiple	8×4	jejunum	Vimentin; Cytokeratins; EMA	Yes	Surgery	8/dead	(33)
1989	82	Female	Retrocecal abscess	NA	Single	12	ileum	Vimentin; Cytokeratins; EMA	Yes	supportive therapy	0.25/dead	(33)
1989	86	Male	abdominal pain and weight loss	NA	multiple	5×4	Sigmoid colon	Vimentin; Cytokeratins	Yes	Surgery	1/dead	(33)
2013	85	Female	dyspepsia and weight loss	CT scan	Single	3×2.5×2.4	jejunum	Vimentin; Cytokeratins; CD56	No	Surgery	3/dead	(13)
1991	46	Male	melena and pallor	NA	Single	14×10×2	jejunum	Vimentin; Cytokeratins; EMA	No	Surgery	6/alive	(26)
1991	57	Male	vomiting, dehydratation and weight loss	NA	Single	6×4.5×2	ileum	Vimentin; Cytokeratins; Desmin	No	Surgery	39/alive	(26)
1991	45	Male	obstruction	NA	Single	3×3×2	ileum	Vimentin; Cytokeratins	Yes	Surgery	0.13/dead	(26)
2013	70	Female	abdominal pain	X-ray	Single	6	jejunum	Vimentin; Cytokeratins; S-100; CD68	No	Surgery	7/alive	(14)
2016	62	Male	abdominal pain and distension	CT scan	Single	12x10	jejunum	Vimentin; Cytokeratins; EMA; PD-L1	No	Surgery	1/dead	(16)
1973	44	Male	Lower GI bleed	NA	Single	5	ileum	NA	Yes	Surgery	48/dead	(10)
2014	56	Female	Vomiting and asthenia	CT scan	Single	10×6.5	jejunum	Vimentin; Cytokeratins;EMA	No	Surgery+chemotherapy	6/dead	(34)
2012	62	Male	abdominal pain	CT scan	Single	15×9×2	ileum	Vimentin; Cytokeratins; EMA; CD68	No	Surgery+chemotherapy	3/alive	(24)
2012	69	Male	Fatigue and melena	Video capsule endoscopy	Single	6×3	jejunum	Vimentin; Cytokeratins	No	Surgery	41/alive	(1)
1996	76	Female	abdominal pain	CT scan	Single	8	jejunum	Vimentin; Cytokeratins; EMA	No	Surgery	2/dead	(35)
2019	61	Male	abdominal pain and melena	Enteroscopy	multiple	6×5×2	ileum	Vimentin; Cytokeratins; EMA; P53	No	Surgery	10/dead	(36)
2017	62	Male	melena	CT scan	multiple	6×4×3	jejunum	Vimentin; Cytokeratins; EMA; S100; CD56	Yes	Surgery	4/dead	(37)
2010	74	Male	abdominal pain	CT scan	Single	4×4×3	jejunum	Vimentin; Cytokeratins; EMA	No	Surgery	6/dead	(38)
2011	58	Male	abdominal pain	X-ray	Single	4×3	ileum	Vimentin; Cytokeratins; EMA	No	Surgery	4/dead	(39)
2019	64	Female	distension	CT scan	Single	25×25	ileum	Vimentin; Cytokeratins	No	Surgery	NA	(40)
1989	62	Male	abdominal pain and weight loss	NA	Single	5	ileum	CEA	Yes	Surgery	20/dead	(20)
1989	38	Female	melena	NA	Single	16×10×4	jejunum	Cytokeratins; CEA	Yes	Surgery+chemotherapy	8/dead	(20)
1989	48	Female	periumbilical mass	NA	multiple	6×4	jejunum	Cytokeratins; CEA; CD15	Yes	chemotherapy	9/dead	(20)
1989	65	Male	abdominal pain	NA	Single	5×5×4	jejunum	Cytokeratins; CEA; CD15	No	Surgery	5/dead	(20)
1989	54	Femal	obstruction	NA	Single	4.5×4	ileum	CD15	No	Surgery	12/dead	(20)
1989	35	Femal	abdominal pain	NA	multiple	7.5	jejunum	CD15	Yes	Surgery	36/dead	(20)

*Take the largest diameter of the tumor if multiple.

**Figure 3:**
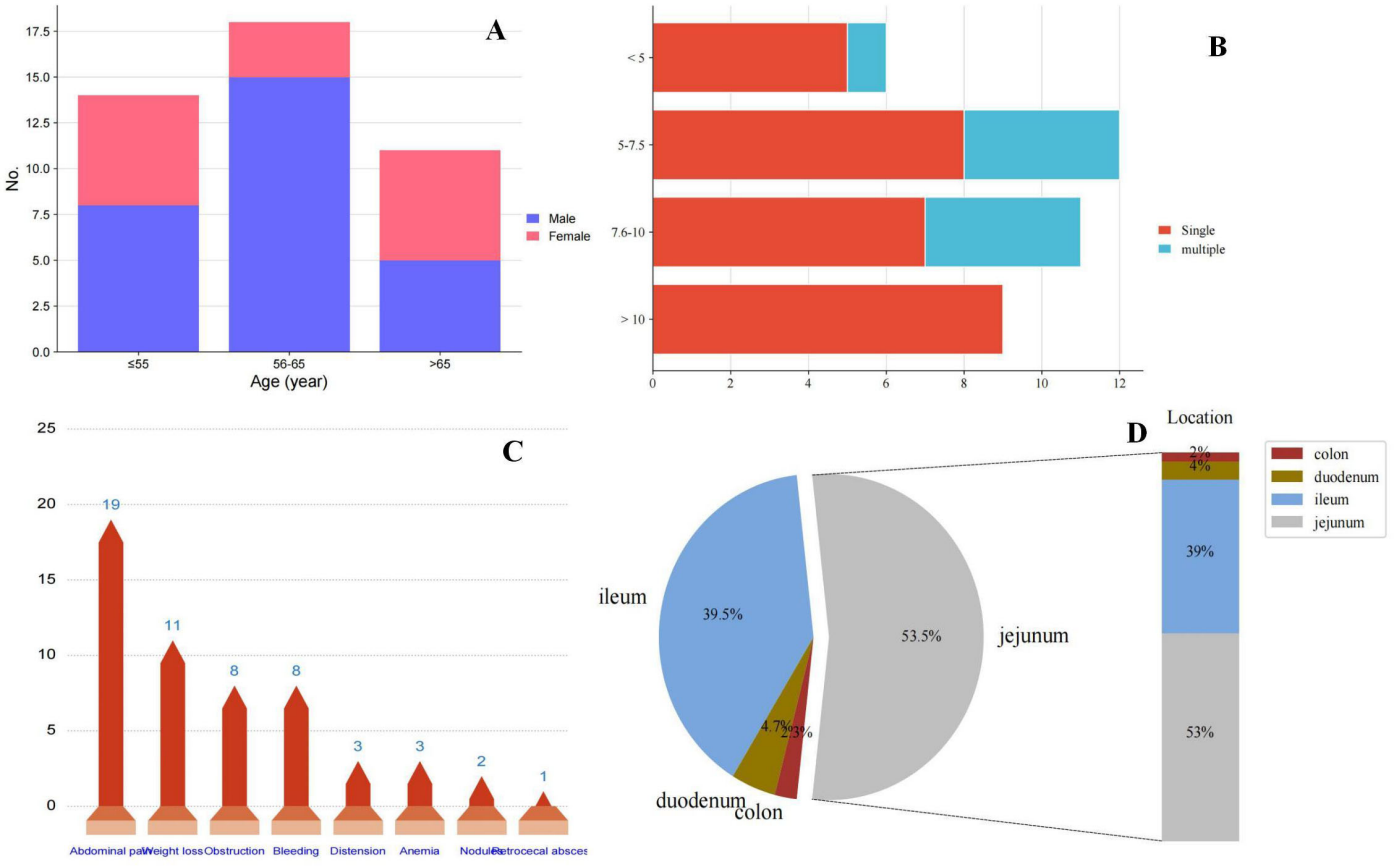
**(A)** Presentation of age at onset of small bowel sarcomatoid carcinoma and gender; **(B)** Presentation of tumor size and single or multiple; **(C)** Distribution of clinical symptoms in patients with small bowel sarcomatoid carcinoma; and **(D)** Distribution of sites of small bowel sarcomatoid carcinoma.

In this cohort of 43 patients, the mean age at presentation was 60.8 years (range 35-85 years). Notably, intestinal SCA was more prevalent in males, with a male-to-female ratio of 1.87:1 (**Figure 3A**), aligning with the ratio of 1.5:1 reported by Reid-Nicholson et al. (11). The clinical manifestations of SCA of the small intestine are nonspecific and largely dependent on the tumor’s location. Common symptoms include abdominal pain, intestinal obstruction, nausea, vomiting, abdominal mass, weight loss, gastrointestinal bleeding, and anemia, with the latter two being most frequent (16). In our literature review, abdominal pain, weight loss, and intestinal obstruction were the predominant clinical features (**Figure 3B**), consistent with previous findings. Notably, our patient presented with gastrointestinal perforation as the initial symptom, a rare occurrence in reported cases. Abdominal CT is a convenient, rapid, and accurate tool for diagnosing small intestine tumors. In our retrospective analysis of 43 patients, 15 were diagnosed via preoperative abdominal CT, while 3 patients with perforation were definitively diagnosed by X-ray. Additionally, 4 patients were diagnosed using small bowel microscopy or videocapsule endoscopy. Hara et al. reported that videocapsule endoscopy is superior to CT in detecting all small bowel lesions (20).

SCA of the small intestine most frequently occurs in the jejunum, followed by the ileum, and is rare in the duodenum and colon. Among the 43 cases we reviewed, 23 (53.5%) were located in the jejunum, 17 (39.5%) in the ileum, 2 (4.7%) in the duodenum, and 1 (2.3%) in the sigmoid colon (**Figure 3C**). Tumor diameters ranged from 2.6 to 25 cm, with most cases presenting as single tumors; the ratio of single to multiple tumors was 30:9. Notably, all SCA with diameters exceeding 10 cm were single lesions (**Figure 3D**).

Histologically, SCA can present as either monophasic or biphasic. Monophasic tumors predominantly feature mesenchymal-like components with minimal or absent epithelioid areas, while biphasic tumors exhibit a mix of epithelioid and mesenchymal-like cells (21), as observed in this case. Diagnosis cannot rely solely on hematoxylin and eosin staining; therefore, a comprehensive panel of immunohistochemical biomarkers is essential for accurate differentiation. Our immunohistochemical analysis revealed strong positive expression of vimentin and cytokeratin (CK), indicating both epithelial and mesenchymal differentiation and thus confirming the diagnosis of SCA. To establish a definitive diagnosis of small bowel SCA, a differential diagnosis is essential. In our study, the absence of HMB45 expression, typically associated with melanoma (22), ruled out primary or metastatic intestinal melanoma. Similarly, the lack of DOG1 expression, a key marker for gastrointestinal stromal tumors (GISTs) (23), excluded the possibility of GIST. Furthermore, CK positivity in our findings effectively excluded mesenchymal-origin malignancies such as fibrosarcoma and liposarcoma. Most sarcomatoid carcinomas exhibit EMA positivity in both epithelial and mesenchymal-like components and approximately 90% of small bowel sarcomatoid carcinomas are positive for vimentin (14). In our literature review, 22 cases (52.38%) were EMA-positive, and 32 cases (76.19%) were positive for vimentin. Given the extended timeframe of the cases reviewed, some earlier reports might lack both immunohistochemical tests, potentially leading to lower observed positivity rates. Positive immunohistochemical staining for the epithelial marker CK is crucial for diagnosing sarcomatoid carcinoma of the small bowel, as it indicates the presence of malignant tumors with an epithelial component (14). In our study, CK positivity was observed in 38 cases (90.48%). Additionally, increased Ki67 labeling index is generally associated with poorer prognosis (14). However, due to its limited reporting in the literature, the prognostic significance of Ki67 requires further validation.

**Figure 4:**
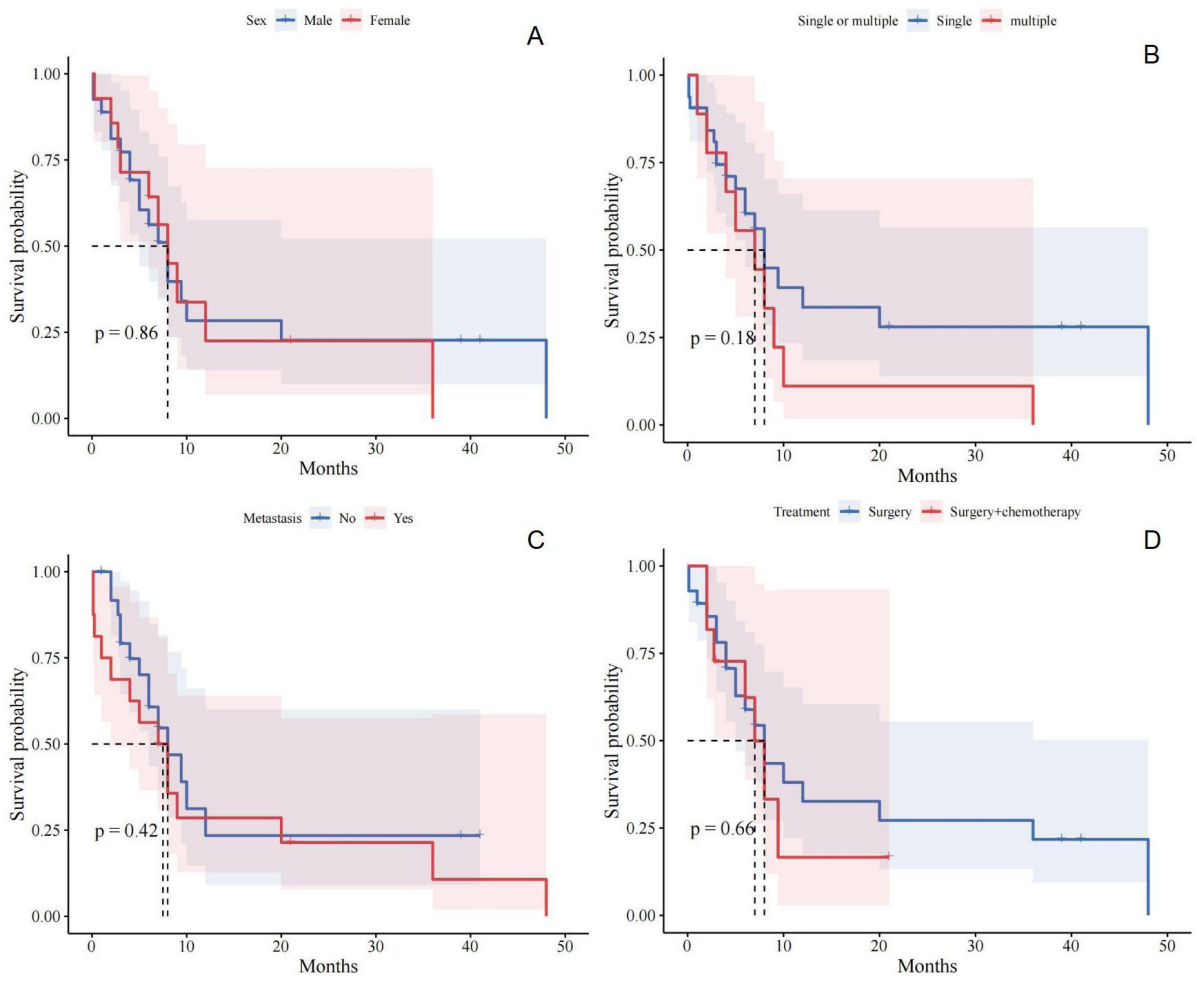
**(A)** Relationship between gender and prognosis of patients with small bowel sarcomatoid carcinoma; **(B)** Relationship between single or multiple and prognosis of patients with small bowel sarcomatoid carcinoma; **(C)** Relationship between distant metastasis and prognosis of patients with small bowel sarcomatoid carcinoma; **(D)** Relationship between different treatment and prognosis of patients with small bowel sarcomatoid carcinoma.

Currently, radical resection is considered the most effective treatment for SCA of the intestine (24, 25). Previous studies indicate that extensive bowel resection around the tumor can improve patient survival (24, 25). In this case, we performed bowel resection where three lesions were located and conducted lymph node dissection. Despite this, the prognosis for patients with SCA of the small intestine remains poor. Notably, our study found that radiotherapy and chemotherapy regimens have not been effective in improving the prognosis for small bowel SCA (19). Overall, 70% of patients with small bowel SCA die within 2 months to 3 years of follow-up (2), with very few surviving beyond 5 years (26). To investigate prognostic risk factors, we conducted a Kaplan-Meier survival analysis of 43 patients. Unfortunately, we found no correlation between gender and prognosis for patients with small bowel SCA (**Figure 4A**). Similarly, the analysis of whether the tumor was solitary or multiple did not reveal any correlation with patient prognosis (**Figure 4B**). Similarly, we assessed the presence of distant metastases in patients, which remained negative (**Figure 4C**). We also compared the effects of surgery alone versus surgery combined with postoperative chemotherapy on patient prognosis. Consistent with previous reports, no significant difference was found between these two treatment options (**Figure 4D**). The rarity of small bowel SCA results in extensive temporal and spatial variability in the analyzed data, compounded by uncertainties in case accuracy, which may introduce bias into our findings. Larger, more robust studies are required to better clarify prognostic risk factors.

In conclusion, SCA of the small intestine is a rare but significant cause of gastrointestinal perforation. Clinical diagnosis is often delayed or inaccurate due to the nonspecific nature of its symptoms. Nevertheless, rare lesions do occur, and it is crucial to consider small bowel malignancy in patients presenting with unexplained acute abdomen. Further large-scale studies are necessary to deepen our understanding of this rare carcinoma and to standardize its treatment protocols.

## Data Availability

The original contributions presented in the study are included in the article/supplementary material. Further inquiries can be directed to the corresponding author/s.
